# Passenger muscle responses in emergency braking events with reclined seating

**DOI:** 10.1038/s41598-023-50918-3

**Published:** 2024-01-02

**Authors:** Lihai Ren, Yuze Kang, Zheng Tan, Chengyue Jiang, Yuanzhi Hu

**Affiliations:** Key Laboratory of Advanced Manufacturing Technology for Automobile Parts, Ministry of Education, Chongqing University of Technology, Chongqing, 400054 China

**Keywords:** Biological techniques, Systems biology, Environmental sciences, Environmental social sciences, Health occupations, Risk factors, Engineering

## Abstract

Emergency braking can generate forward displacement that may influence the effectiveness of protection in collisions, especially for passengers. The development of automated vehicles has enabled the diversification and rationalization of sitting positions, including reclined seating. However, the passenger response in pre-crash scenarios in reclined seating differs from that in standard seating, which poses different requirements for biofidelic human body models (HBMs) to evaluate passenger injuries in collisions. This study conducted emergency braking trials in vehicles at an initial velocity of 80 km/h. Five volunteers were exposed to approximately 1 g manual emergency braking (MEB), and the muscle responses at the front passenger seat with backrest angles of 25°, 45°, and 65° were recorded. The electromyography obtained from 14 muscles of the neck, torso, and lower extremity were normalized using maximum voluntary contractions (MVCs). In the quiet sitting phase, the activity levels were low (< 5% MVC) in all muscles for the three sitting positions. During emergency braking, the muscles are activated to restrict the body motion. There were differences in muscle amplitude and onset time in different backrest angles, with higher muscle activity levels in most muscles in a reclined sitting position. In particular, the sternocleidomastoid, rectus abdominis, and vastus medialis showed different patterns in the peak and steady-state phases. We found that the tibialis anterior was consistently activated at a lower level in all sitting postures (< 8% MVC), which indicates limited support of the shank for the body. The data provided in the paper are presented in corridors and intended for use in the development and validation of HBMs with active muscle models to simulate evasive maneuvers that potentially occur before a crash in the reclined sitting position.

## Introduction

Road traffic injuries are a public health issue with social influence. The Global status report on road safety 2018 launched by WHO, highlights that the annual number of road traffic deaths exceeds 1.3 million and is increasing^1^. Road traffic crashes have become one of the leading causes of death, predicted to reach the fifth place in the year 2030^2^. Therefore, road safety remains a serious topic.

Considerable research effort has been devoted to the development of safety technologies in vehicles to avoid crashes or mitigate crash severities. The use of human body models (HBMs) for evaluation is currently an important research method. Some HBMs with muscle controllers can simulate the occupant response in pre-crash scenarios, such as the Total Human Model for Safety, the Active Human Body Model, and the Global Human Body Models Consortium^3–5^. However, to effectively develop and validate biofidelic HBMs, biomechanical data for occupants in different representative potential pre-crash situations are essential.

Braking is an important collision avoidance behavior by drivers in response to potential crashes^6,7^. However, braking will cause the forward displacement of passengers, which changes the sitting posture and body position. Several studies have investigated the impact of braking maneuvers on the occupant kinematics^8–10^. These previous studies quantified the changes in occupant posture during the braking maneuver. Kirschbichler et al.^11^ and Huber et al.^12^ found that emergency braking significantly impacted the forward displacement of the head and torso. Antona et al.^13^ showed that braking would subject the occupants to higher loads and a larger forward traveling distance of the torso during the collision. Furthermore, the response of occupants during the pre-impact braking indicates that muscle contraction is an important factor that affects the forward displacement^14^. These results demonstrate that the braking maneuver and muscle contraction can change the kinematics of the occupant. The braking force can also exert a higher load on the neck muscles in a rear-end collision, which may increase the risk of neck injury^15^. These results are crucial for the development of safety technologies in vehicles.

Several studies have provided quantified data on the occupant muscle response under different loads. Ghaffari et al.^16^ and Ghaffari et al.^17^ examined the passenger muscle responses in lane change and lane change with braking maneuvers in both male and female volunteers using standard and reversible pre-pretensioner seat belts, and they found a higher average activation level in the lumbar paravertebral muscles and neck muscles. Similarly, Östh et al.^18^ and Ólafsdottir et al.^19^ provided the kinematics and muscle responses of the drivers and passengers while braking, respectively. Graci et al.^20^ compared the differences in occupant kinematics and muscle responses between autonomous emergency braking (AEB) and manual emergency braking (MEB). The objective of these studies is to provide data that enable the development and validation of HBMs. All of these studies used seats with a standard backrest angle. Kang et al.^21^ provided the muscle responses of a passenger in different backrest angles exposed to AEB, where the maximum seat angle relative to the vertical was 32° in their trial. However, there is a lack of studies on the passenger muscle responses in a reclined seat.

The development of automated vehicles has rationalized diverse seating positions, including different reclined sitting positions. A survey reported that passengers desired that the seat functions include being able to recline to a horizontal position^22^. Laakmann et al.^23^ and Matsushita et al.^24^ showed that the reclining posture could increase the risk of injury for the occupants, especially to the head and neck. Another study found that starting from 117° relative to the horizontal, when the seatback angle increased, the value of Head Injury Criterion also increased^25^. Reclined seating can make the head rotate during a collision, which increases the risk of neck injury^26^. In addition, some studies have shown that when the seatback angle increases, the likelihood of submarining increases^27,28^.

The HBM in reclined seating and muscle responses during braking must be validated with volunteer data, including quantifiable muscle contractions for passengers. The present article provides the muscle responses of volunteers in the front passenger seat at different seatback angles during MEB.

## Methods

The kinematics and electromyography (EMG) responses of the front-seat passenger volunteers were recorded in 1 g MEB events in a test vehicle, which was driven on the motor vehicle inspection road, a closed course. Three types of test cases were conducted for the front-seat passenger volunteers. Five volunteers participated in the trials, and each volunteer repeated the trial three times at each backrest angle. In all test cases, the volunteers were restrained with an automatic locking seat belt that did not have electronic pretensioning. All volunteers performed maximum voluntary contractions (MVCs) for the EMG normalization. All volunteers agreed to participate in this study and gave their written informed consent before testing. They have allowed their information and data to be used for public publication. The study protocol was reviewed and approved by the Ethical Review Board at the Chongqing University of Technology. The experiment followed the Declaration of Helsinki, and the methods were conducted in accordance with relevant guidelines and regulations.

### Volunteers

Five healthy young male volunteers (Table 1) without neuromuscular conditions or a history of neck pain were included in this study. Volunteers were selected based on the 50th percentile height and weight criteria for adult male, and all volunteers were similar in age and shape; the average age, height, weight, and body mass index (BMI) were 24.2 ± 0.37 years, 175 ± 2.45 cm, 71.6 ± 2.73 kg, and 23.4 ± 0.43 kg/m^2^, respectively. All volunteers were prohibited from drinking alcohol and engaging in vigorous exercise before testing.

**Table 1:** Volunteer descriptive data.

	Age (years)	Height (cm)	Weight (kg)	BMI (kg/m^2^)
Volunteer 1	24	176	72	23.2
Volunteer 2	24	175	74	24.2
Volunteer 3	24	173	69	23.1
Volunteer 4	24	179	75	23.4
Volunteer 5	24	172	68	23.0

### Instrumentation

The MEB test cases were performed in a Sport Utility Vehicle. The vehicle was fitted with a ventilation disk brake and noise-canceling tires (255/50 R20). The seats were covered in leather upholstery, located in the middle position of the front-to-back travel, adjusted to the lowest height, and equipped with an automatic locking seat belt. An accelerometer was mounted on the floor below the B-pillar to measure the longitudinal vehicle speeds and accelerations at 1000 Hz.

**Figure 1:**
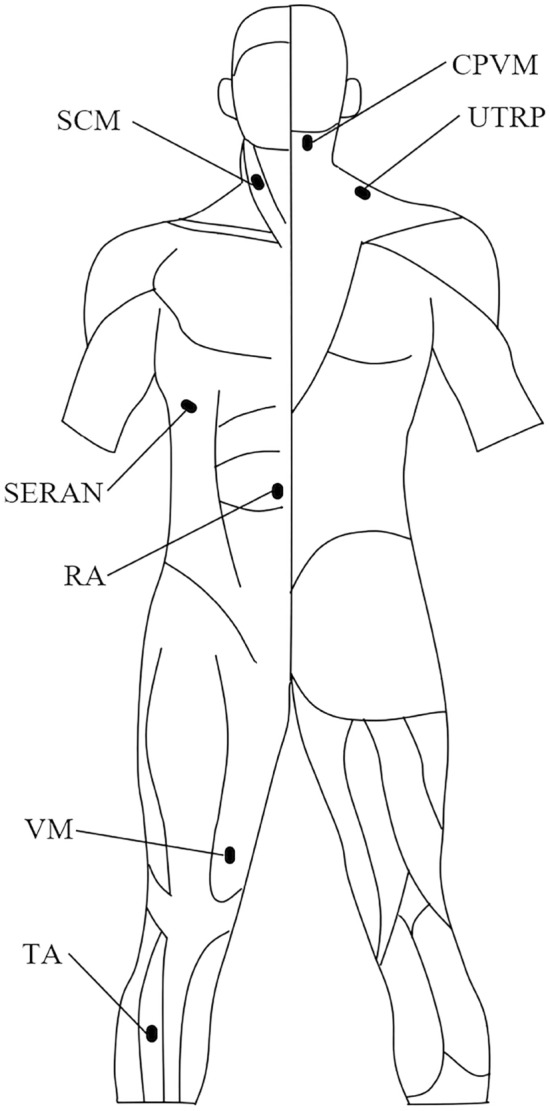
Electrode placement on the anterior and posterior sides of the body shown to the left and right. The figure was created using Microsoft Office PowerPoint version 2021 (https://www.microsoftstore.com.cn/software/office).

The electrode placement to measure the EMG was referenced from Ghaffari et al.^16^ and SENIAM^29^. Fourteen surface electrodes were placed bilaterally on the sternocleidomastoid (SCM), cervical paraspinal muscles (CPVM), upper trapezius (UTRP), serratus anterior (SERAN), rectus abdominis (RA), vastus medialis (VM), tibialis anterior (TA) to measure EMG activity, as shown in Fig. 1. EMG (Tringo Wireless EMG System, Delsys, Inc., Boston, MA, USA) was recorded at a sampling rate of 1111 Hz. Before the electrode placement, the skin had to be shaved, abraded with scrub cream, and wiped with 75% alcohol.

**Figure 2:**
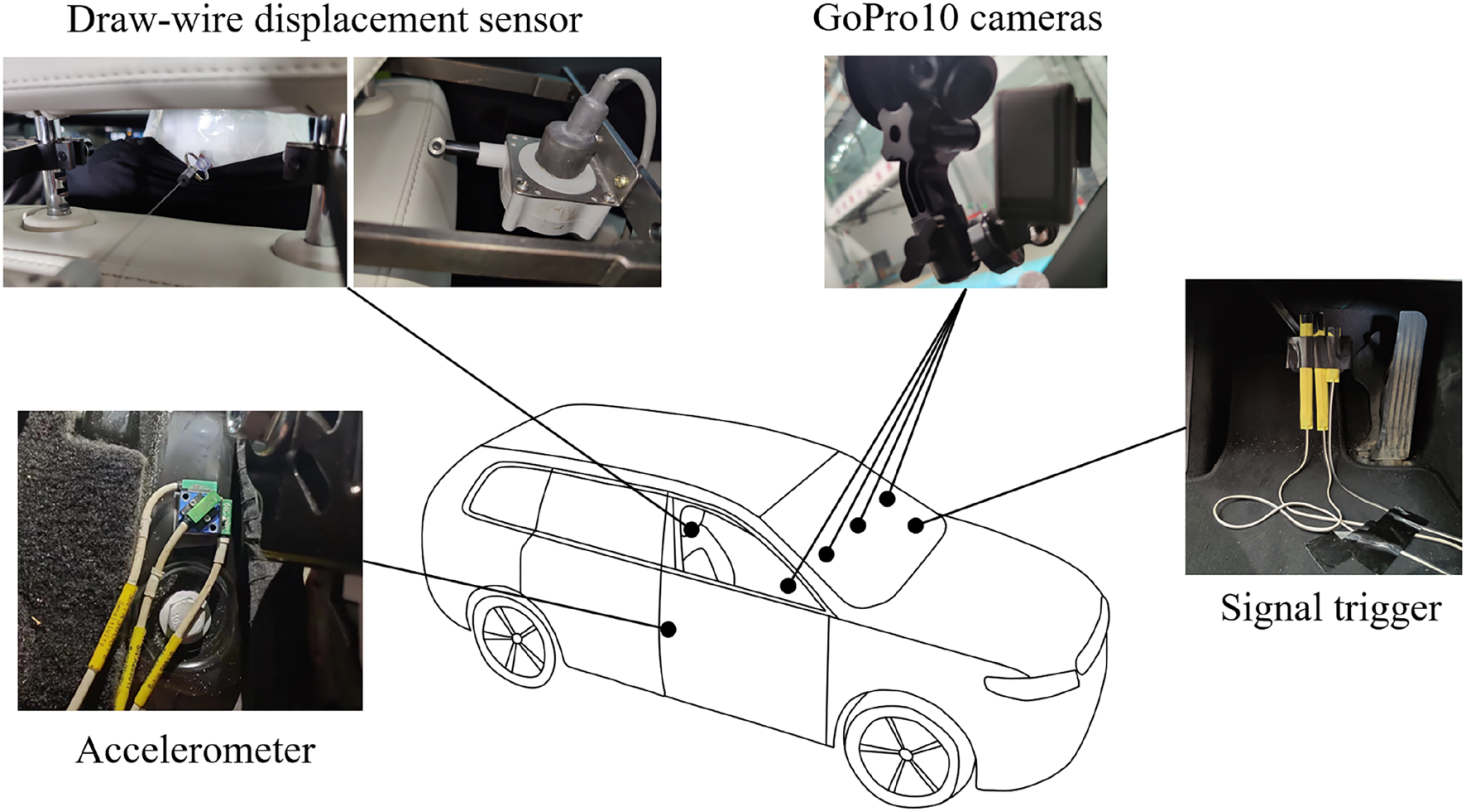
Image showing the installation locations of all instruments. The figure was created using Microsoft Office PowerPoint version 2021 (https://www.microsoftstore.com.cn/software/office).

The head displacement of the volunteers was captured using four high-definition motion cameras GoPro (HERO 9 Black, GoPro, Inc., San Mateo, CA, USA) at a sampling rate of 120 Hz. The cameras were mounted on the right side, right front side, front side, and left front side of the front-seat passenger. Calibrated the cameras using a black-and-white checkerboard pattern. The markers with a diameter of 15 mm were affixed on the left side of the head, and scales were affixed on the B-pillar and bottom edge of the window. Tracked the markers on the head in the video using OpenCV and employed the binocular vision algorithm to obtain spatial coordinates, thus measuring the passengers’ head displacement. The T1 displacement was measured using a draw-wire displacement sensor (SP2-50, TE Connectivity, Inc., Berwyn, PA, USA), which was installed on the headrest position and connected to the neck. Attached the end of the draw-wire displacement sensor to a suction cup, and used tape to secure the suction cup to the soft plastic collar worn around the volunteer’s neck. The T1 displacement was obtained by measuring the draw-wire displacement during braking. Figure 2 shows the installation locations of all instruments.

All instrument recording start times were synchronized through a signal trigger, which was placed on the brake pedal. The signal was triggered when the driver began to depress the brake pedal. The initial time point for sensor signals was recorded to ensure synchronization of different data.

### Test procedure

Five volunteers underwent 45 MEB trials in the front passenger seat in total, including three different backrest angles of 25°, 45°, and 65°. The volunteers received special training before testing to familiarize them with the test procedures and instructions. The volunteers were instructed to relax and keep their feet placed on the floor, with hands resting on their thighs and heads leaning against the headrest. During the testing, no instructions or interventions were given to the volunteers to ensure that they remained in a relaxed state. The vehicle was driven by a professional tester; the initial velocity in all test cases was approximately 80 km/h. Before the MEB maneuver, the vehicle maintained a constant initial speed to measure the EMG in quiet sitting. Finally, the driver suddenly pressed the brake pedal without informing the volunteer in advance. This method can keep the volunteers unaware of the moment and location for initiating braking action, which can to prevent anticipatory behavior. Each volunteer underwent nine MEB trials in the front passenger seat, i.e., three repeated trials for each seatback angle. The study by Ólafsdottir et al.^19^ indicated that the reduction in head displacement and muscle activity due to repeated trials was not universal, indicating that the impact on the results is limited. The testing sequence followed an increasing order of backrest angles, starting with three tests at 25° and concluding with three tests at 65°. After each repetition, there was a brief break of approximately 10 min to ensure that the muscles did not become fatigued, and data were recorded.

**Figure 3:**
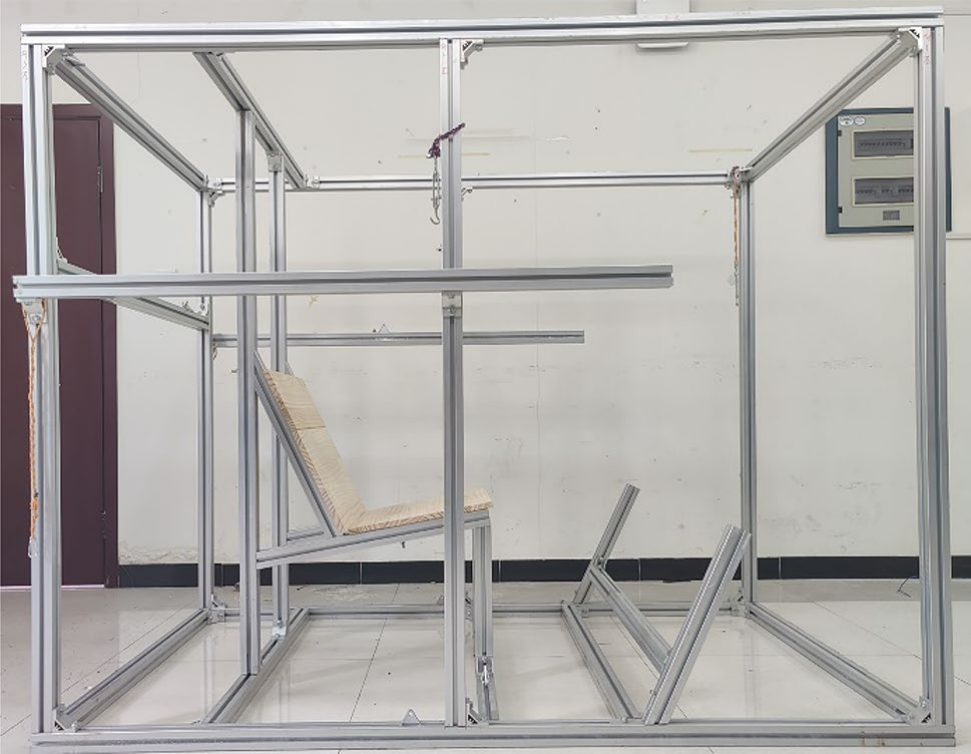
Customized test bench.

**Figure 4:**
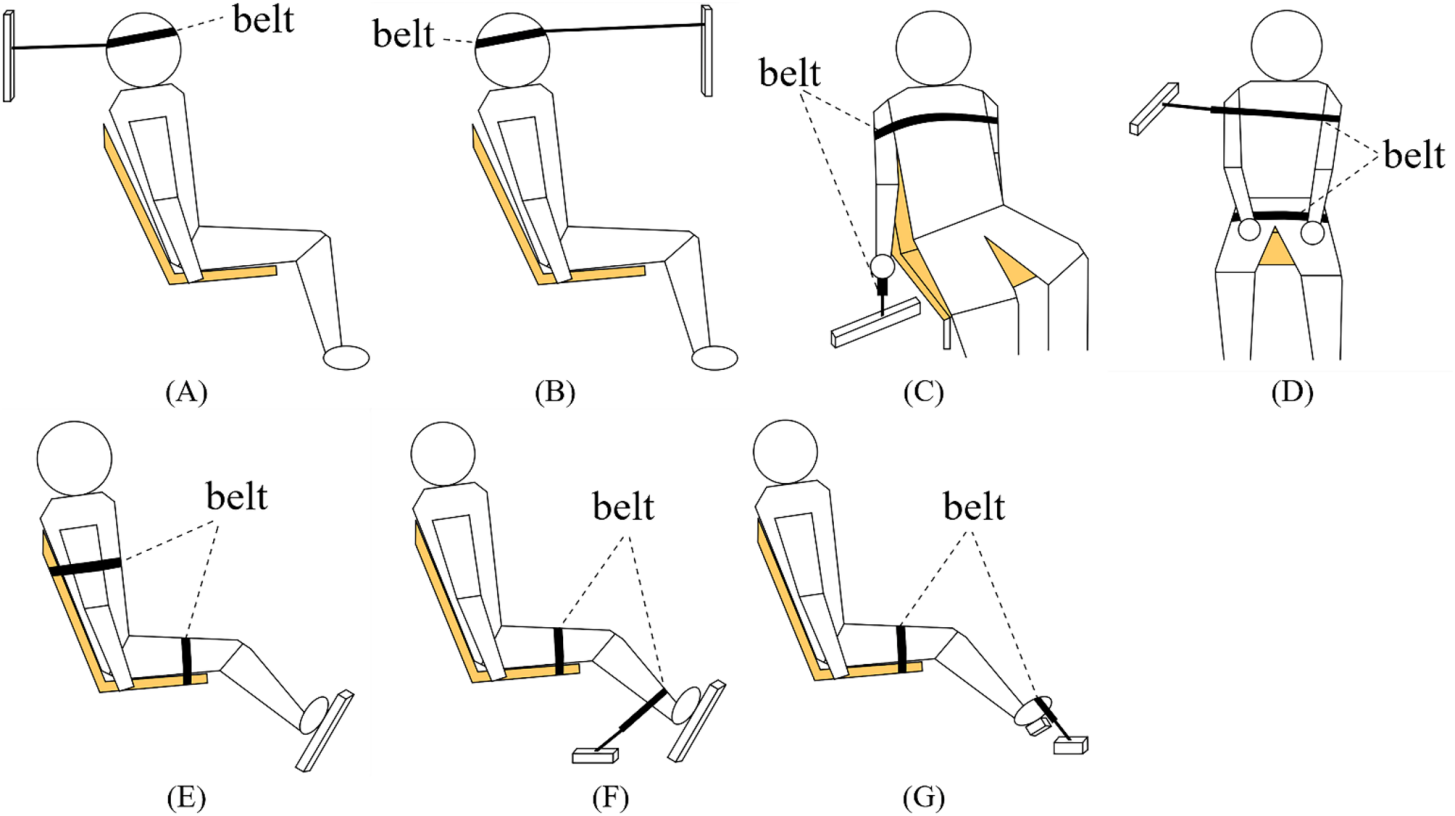
Postures and instructions for the MVCs. (**A**) Cervical flexion. No support from feet; arms along the side. (**B**) Cervical extension. No support from feet; arms along the side. (**C**) Shoulder lift. No support from feet; arm restricted just above the hand. (**D**) Lumbar side bending. Feet supported; thighs supported by the belt; and hands on the thighs. (**E**) Lumbar flexion. Feet supported; thighs supported by the belt; and arms along the side. (**F**) Knee extension. Thighs supported by the belt; arms along the side; and leg restricted just above the ankle. (**G**) Ankle flexion. Thighs supported by the belt; arms along the side; and leg restricted just above the foot. The figure was created using Microsoft Office PowerPoint version 2021 (https://www.microsoftstore.com.cn/software/office).

The volunteers performed MVCs on a customized test bench (Fig. 3). The EMG data were recorded during maximum muscle tension in isometric conditions. The seat of the test bench was made of wood; the backrest angle was 25° with a 10° incline of the seat cushion relative to the horizontal plane. The volunteers were instructed to adopt a posture that resembled the typical sitting posture in a vehicle and to ensure that their lower extremities felt comfortable. The procedure suggested by Östh et al.^18^ and Ghaffari et al.^16^ was adopted. The volunteers were asked to contract approximately 50% of the maximum for 3 s, followed by an increase to a maximum for 3 s. Each muscle was tested three times with approximately 30 s of rest between each repetition and approximately 3 min of rest between MVC tests for different muscles. The series of MVC tests are described in Fig. 4.

### Data analysis

Longitudinal vehicle accelerations were low-pass filtered using a second-order Butterworth filter with the cutoff frequency set to 6 Hz^20^. The average and standard deviation (SD) of the acceleration curves for each braking trial were calculated to examine the repeatability of the maneuver. From the longitudinal acceleration curve amplitude of the braking, three phases were defined^20^. The braking maneuver onset was defined as 5% of the maximum longitudinal acceleration. The steady-state acceleration start was defined as the first time when the acceleration exceeded 85% of the maximum. The steady-state acceleration end was defined as the last time when the acceleration exceeded 85% of the maximum.

The coordinate data for head displacement was presented in a vehicle-fixed coordinate system defined in accordance with ISO8855, where the positive X direction is forward, and the positive Z direction is upward. The head displacement was calculated by transforming marker displacement on the left side of the head using binocular stereo vision.

The raw EMG signals were band-pass filtered using a fourth-order Butterworth filter with the cutoff frequency set to 10–350 Hz. Because the electrocardiogram (ECG) signal influenced EMG data that were collected in the torso, the lower cutoff frequency was increased to 50 Hz for the EMG data recorded from SERAN and RA^19^. A 50 Hz notch filter was used to eliminate the noise generated by the AC power supply. Finally, the signal was subjected to full-wave rectification and smoothed using a 45-sample (approximately 40 ms) moving root mean square (RMS) window^19^. The EMG was normalized with the maximum of all three MVCs for the corresponding muscles. Furthermore, the muscle activity onset time was defined as the beginning of a 55-sample (approximately 50 ms) window, for which the average EMG data were 2.5 SDs greater than the average of the EMG data in the quiet sitting as previously proposed^16,30^.

After the EMG was calculated for each trial, outliers that were 3 SDs greater than the mean value for the same loading scenario were removed. Shapiro-Wilk test of normality was performed on the mean EMG values and onset time for each loading scenario. The results showed that some of the muscle data points were normally distributed, while some others were not. Therefore, a nonparametric Friedman test with a 5% significance level was applied to the EMG data. Because the Friedman test requires a balanced dataset, all outliers and imbalanced data were removed from the statistical analysis. The differences in mean EMG values, which were analyzed using the aforementioned methods, were assessed in the time interval of 1.5–2 s during the steady-state braking phase.

## Results

The vehicle dynamics and EMG corridors were represented using means ± SD. The zero point in the charts indicates the start time of the instrument recording, which is when the driver began to depress the brake pedal.

Few EMG signals had outliers. For Volunteer 2, the instrument trigger time was advanced once and delayed once in the 25° and 45° trials, respectively. For the remaining outliers, no certain explanation could be identified. The cause could be poorly attached electrodes or pressure artifacts. Those outliers are not shown in any graphs.

### Vehicle dynamics

**Figure 5:**
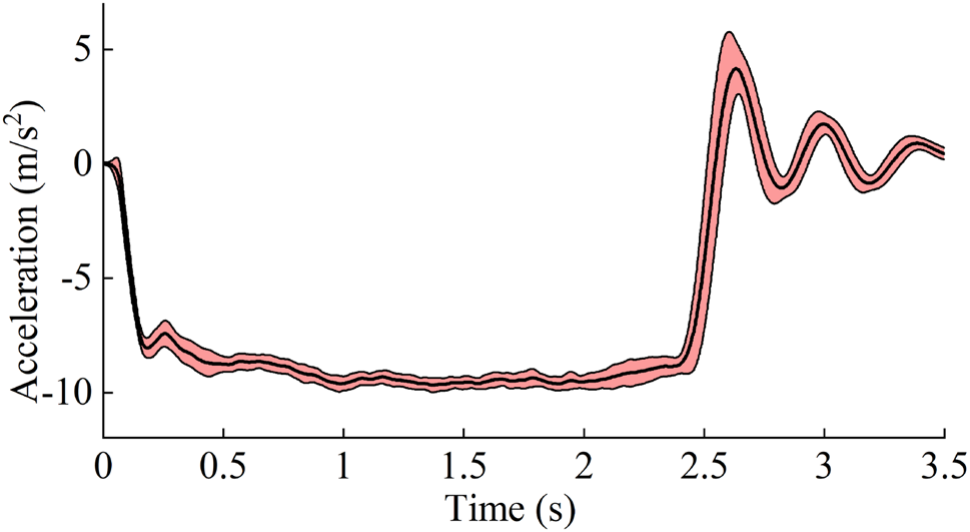
Average ± SD of the vehicle acceleration.

Figure 5 shows the average acceleration pulses for all test cases. In the trials, the average jerk was 3.24 g/s from the braking maneuver onset time to the steady-state acceleration phase. The average acceleration during the steady-state phase was 9.27 ± 0.15 m/s^2^ with a duration of 2.03 ± 0.15 s.

### Kinematics

**Figure 6:**
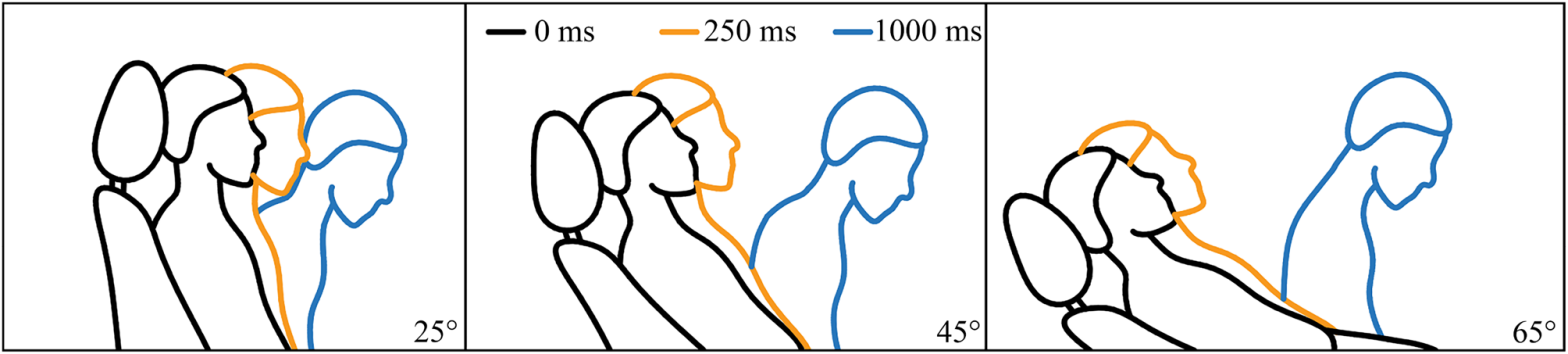
The body movement at 0 ms, 250 ms, and 1000 ms at backrest angles of 25°, 45°, and 65°. The figure was created using Microsoft Office PowerPoint version 2021 (https://www.microsoftstore.com.cn/software/office).

**Figure 7:**
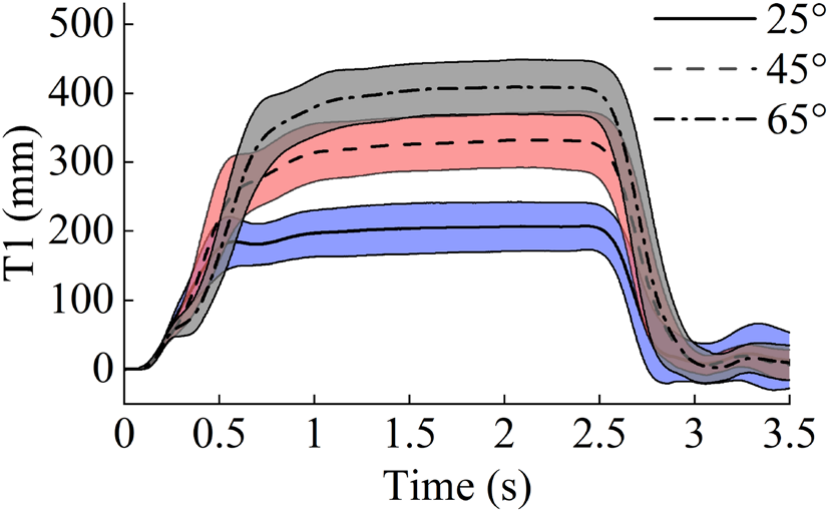
Average ± SD of the T1 displacement.

**Figure 8:**
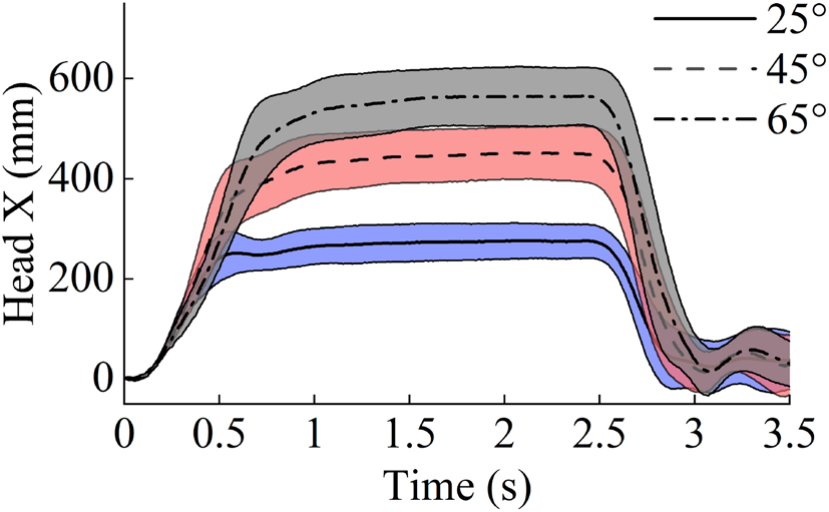
Average ± SD of the head X displacement.

Figure 6 shows the sequence of a typical body movement at 0 ms, 250 ms, and 1000 ms, with backrest angles of 25°, 45°, and 65°. Figures 7 and 8 show the mean and SD of T1 and head X displacement in the test cases at 25°, 45°, and 65°. In all test cases, the average displacement of the volunteer increased when the seatback angle increased. In the test cases at 25°, 45°, and 65°, Table 2 shows the mean and SD peak of T1 and head X displacement.

**Table 2:** Mean and SD peak of T1 and head X displacement for all test cases.

	25°		45°		65°	
	Mean	SD	Mean	SD	Mean	SD
T1 (mm)	207.1	34.3	332.5	40.1	409.2	39.5
Head X (mm)	275.9	35.5	451.8	52.0	564.8	57.4

### EMG response

**Figure 9:**
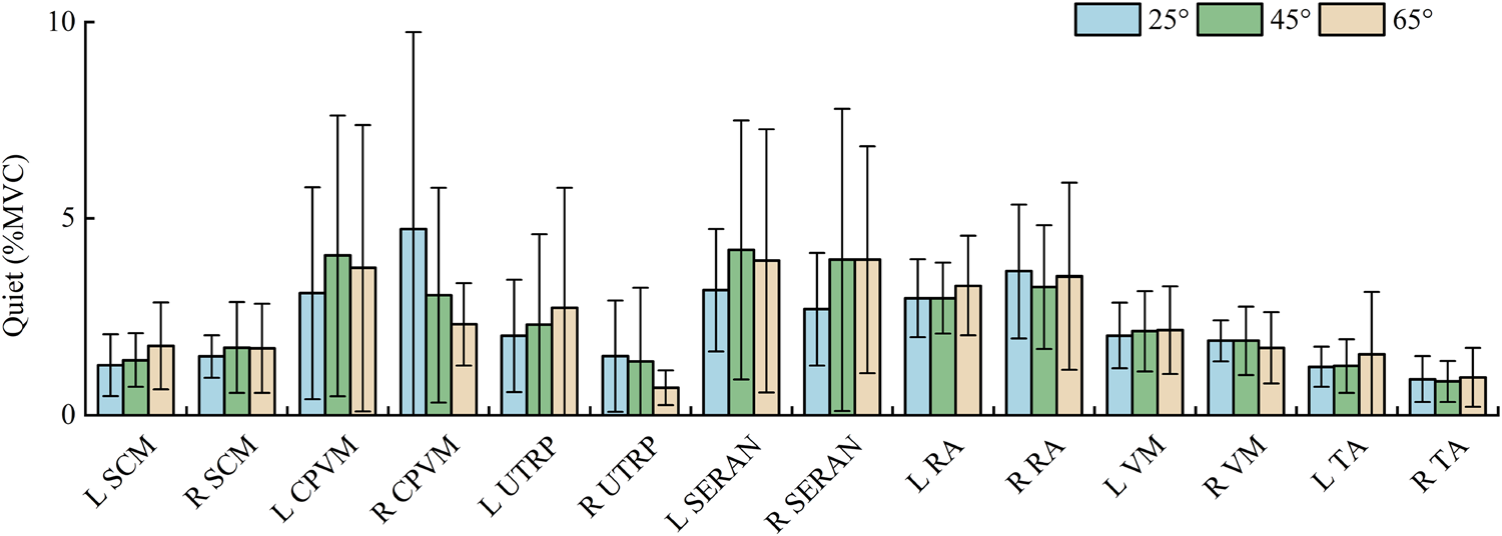
Average and SD of the EMG during the quiet sitting phase at 25°, 45°, and 65° backrest angles.

In the quiet sitting phase, the average activation of muscles was less than 3% MVC except for CPVM, SERAN, and RA in all angles. All muscles were activated less than 5% MVC on average (Table 3). The similar muscle activation levels during the quiet sitting phase indicate that the volunteers were in similar muscle states in different sitting postures before the braking maneuver. Figure 9 shows the average ± SD and peak of the EMG during the quiet sitting phase at 25°, 45°, and 65° backrest angles.

**Table 3:** Mean, SD and peak of the muscle activity as % MVC in quiet sitting for all test cases.

Muscle	Side	25°			45°			65°		
		Mean	SD	Peak	Mean	SD	Peak	Mean	SD	Peak
SCM	L	1.27	0.78	2.08	1.40	0.68	2.05	1.76	1.10	2.60
	R	1.49	0.54	2.61	1.72	1.15	2.90	1.70	1.13	2.94
CPVM	L	3.10	2.69	5.06	4.05	3.57	6.07	3.74	3.64	5.68
	R	4.72	5.02	9.82	3.05	2.73	4.68	2.31	1.05	3.87
UTRP	L	2.02	1.43	3.76	2.30	2.29	5.80	2.73	3.05	6.82
	R	1.50	1.41	2.77	1.37	1.87	3.47	0.70	0.44	0.99
SERAN	L	3.17	1.55	5.36	4.20	3.29	11.62	3.92	3.34	10.47
	R	2.69	1.43	4.80	3.95	3.84	8.47	3.95	2.88	5.42
RA	L	2.97	0.99	4.54	2.97	0.90	4.00	3.29	1.26	4.37
	R	3.65	1.70	5.04	3.25	1.57	4.12	3.53	2.37	4.60
VM	L	2.02	0.83	2.65	2.13	1.02	2.87	2.16	1.11	2.94
	R	1.89	0.52	2.44	1.89	0.87	2.58	1.71	0.90	2.49
TA	L	1.23	0.51	1.54	1.25	0.68	1.73	1.55	1.58	2.29
	R	0.92	0.58	1.30	0.86	0.52	1.21	0.96	0.75	1.21

**Figure 10:**
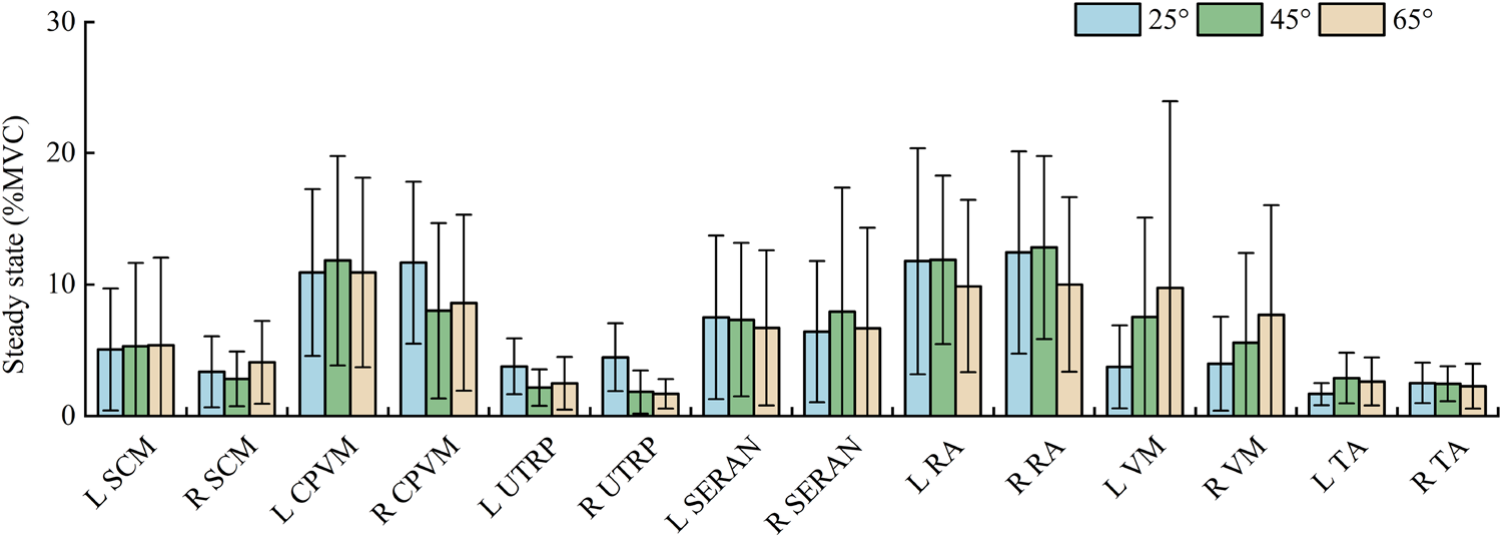
Average and SD of the EMG during the steady state at 25°, 45°, and 65° backrest angles.

**Figure 11:**
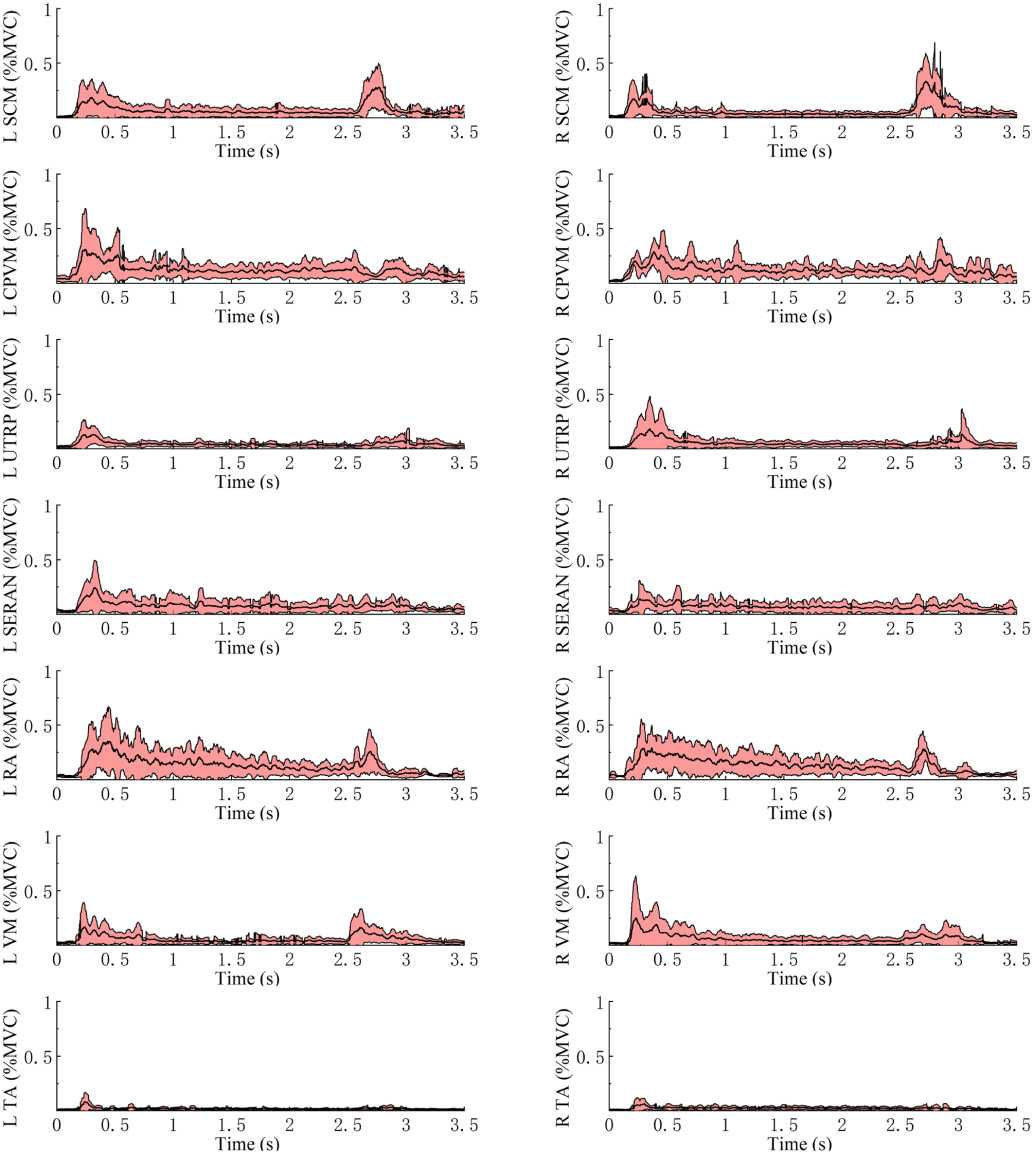
Average ± SD EMG response normalized to MVC for muscles in MEB on both sides for the 25° backrest shown to the left and right.

**Figure 12:**
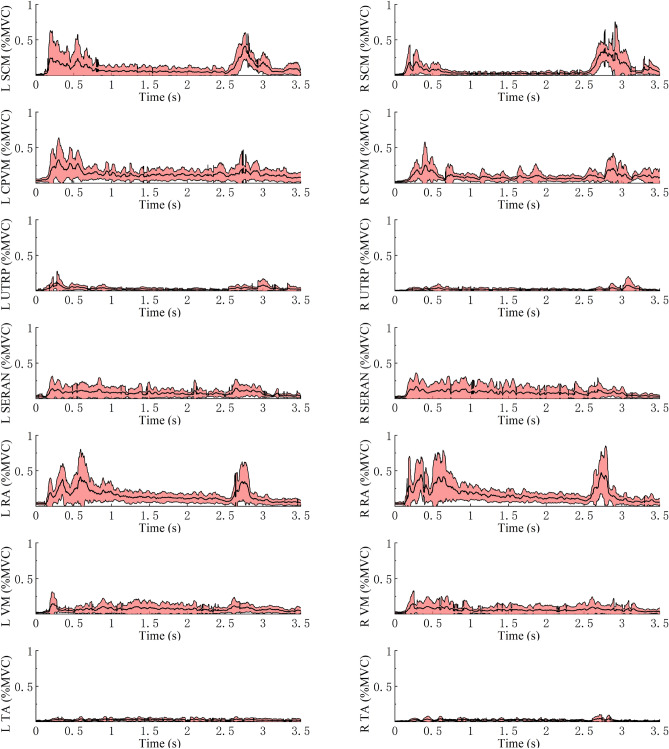
Average ± SD EMG response normalized to MVC for muscles in MEB on both sides for the 45° backrest shown to the left and right.

**Figure 13:**
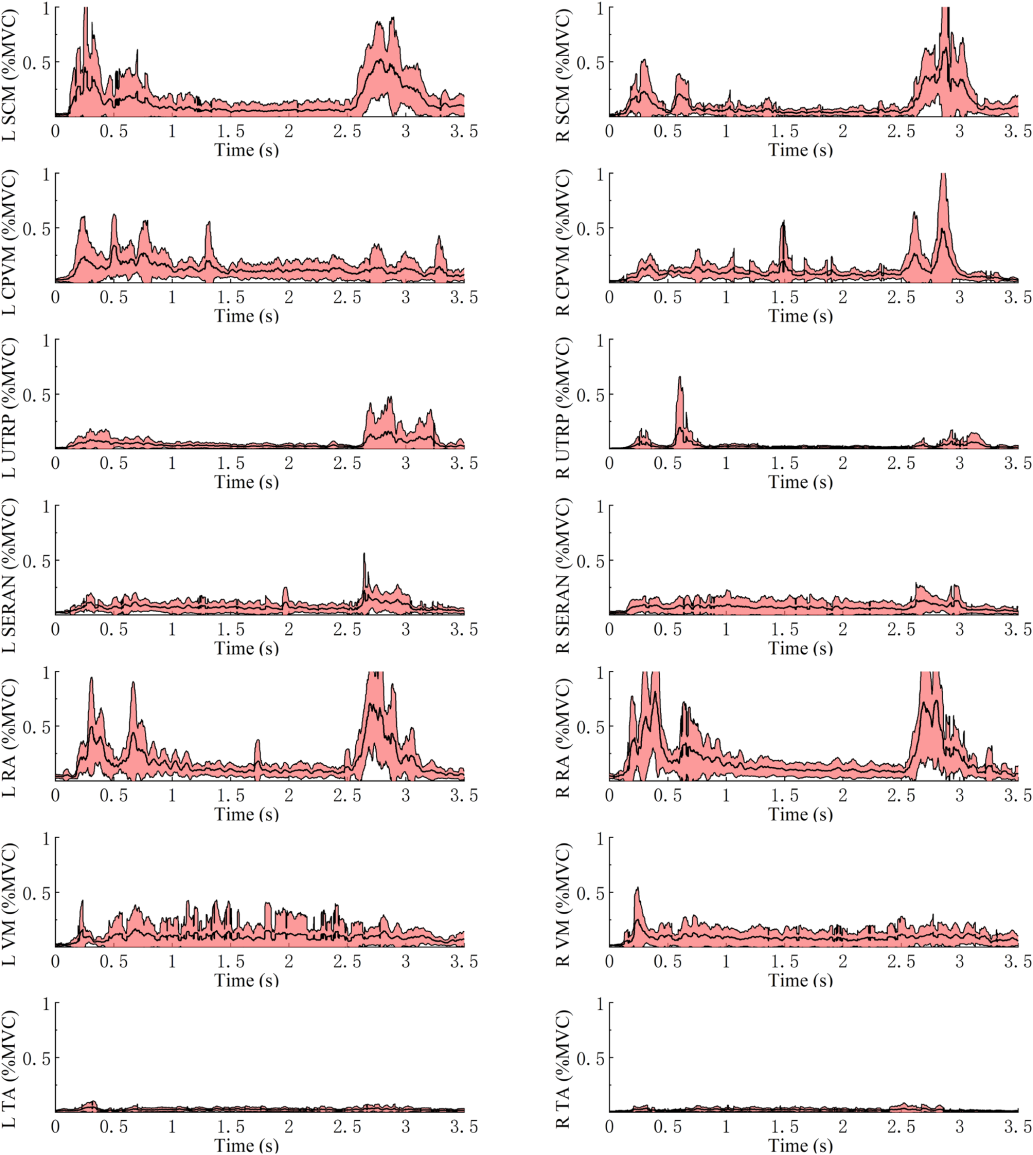
Average ± SD EMG response normalized to MVC for muscles in MEB on both sides for the 65° backrest shown to the left and right.

Table 4 shows the mean, SD, and peak of muscle activity in steady-state braking for both sides of the body, as illustrated in Fig. 10. Figures 11, 12 and 13 show the average EMG data for the muscles on the left and right sides. In steady-state braking, the muscle activity did not significantly differ between the left and right sides in all test cases. The UTRP muscles had statistically significant differences between 25° and 45° on both sides of the body and between 25° and 65° on the right side (*P* < 0.05). In contrast, other muscles showed no significant differences in the steady-state braking phase in any test case.

**Table 4:** Mean, SD and peak of the muscle activity as % MVC in steady state for all test cases.

Muscle	Side	25°			45°			65°		
		Mean	SD	Peak	Mean	SD	Peak	Mean	SD	Peak
SCM	L	5.06	4.63	14.77	5.31	6.33	18.78	5.40	6.64	19.47
	R	3.36	2.70	9.04	2.84	2.08	6.07	4.08	3.15	9.03
CPVM	L	10.91	6.34	20.19	11.81	7.96	22.56	10.90	7.20	19.52
	R	11.66	6.16	20.54	7.99	6.65	14.93	8.60	6.68	19.49
UTRP	L	3.77	2.12	6.83	2.17	1.39	5.63	2.48	2.00	6.57
	R	4.46	2.57	7.59	1.83	1.63	5.55	1.68	1.13	3.52
SERAN	L	7.49	6.22	19.53	7.32	5.82	19.53	6.69	5.90	19.21
	R	6.41	5.36	18.26	7.92	9.44	28.32	6.67	7.62	23.05
RA	L	11.76	8.59	28.14	11.87	6.41	20.99	9.88	6.54	18.76
	R	12.42	7.68	25.19	12.80	6.96	24.82	10.01	6.64	18.33
VM	L	3.73	3.15	9.50	7.52	7.56	21.32	9.74	14.21	45.05
	R	3.97	3.57	10.54	5.57	6.80	20.42	7.67	8.36	24.03
TA	L	1.67	0.83	2.56	2.89	1.92	6.36	2.62	1.83	5.65
	R	2.52	1.54	4.30	2.46	1.33	4.55	2.27	1.70	4.97

For all volunteers who participated in the trial, the average muscle activity rapidly increased in all muscles except for TA after the driver began to depress the brake pedal (Figs. 11, 12 and 13). The SCM and RA showed higher EMG peaks at 45° than at 25°; this pattern was also observed at 65° compared with 45°. The left- and right-side TA activity levels were low in all conditions: the maximum average activity levels were less than 7% MVC and less than 3% MVC in the steady-state phase. For the three conditions, the CPVM and RA activation levels were higher in the steady-state phase with an average of 8–13% MVC (Table 3). Furthermore, higher activation levels of the neck muscles (SCM, CPVM, and UTRP) were observed after the driver began to depress the brake pedal, but UTRP was relatively lower.

For different backrest angles, the VM showed higher average activity levels in the reclined sitting posture than in the standard sitting posture during the steady-state phase: the activity level was higher at 65° than at 45° and higher at 45° than at 25°. In contrast, no similar trend was observed in the average activation levels during the steady-state phase of the other muscles. The differences in these muscles for different backrest angles were mainly observed in amplitude after the onset of braking.

### EMG onset time

**Figure 14:**
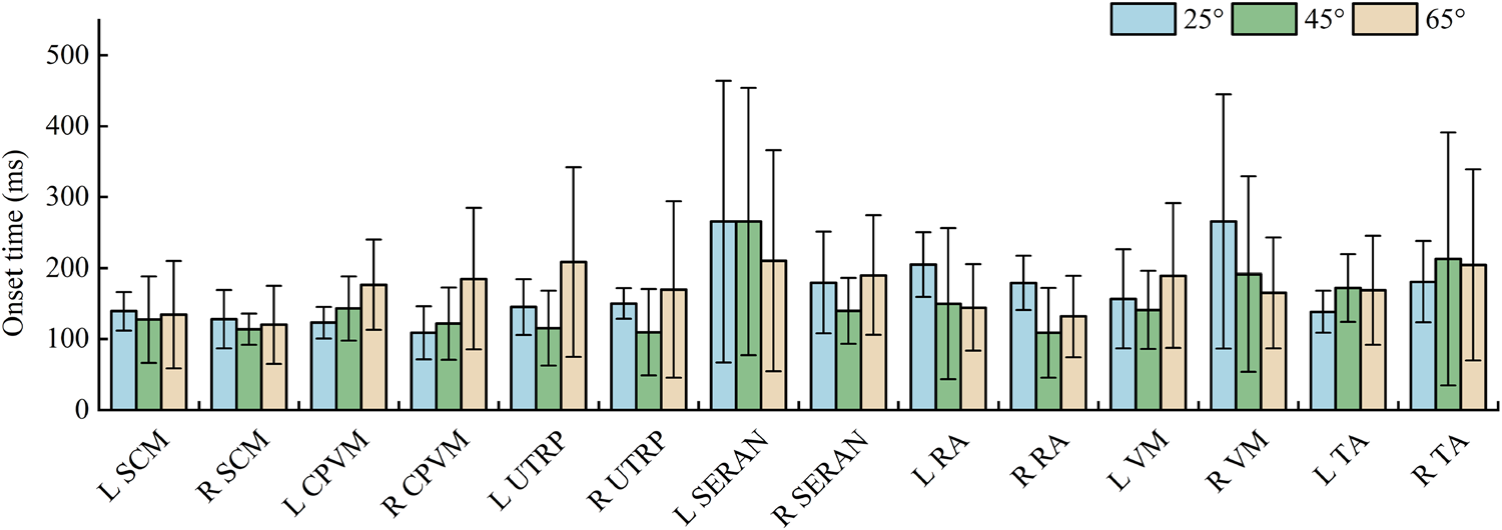
Average and SD of the EMG onset time during emergency braking at 25°, 45°, and 65° backrest angles.

The average EMG onset time for MEB differed among the 25°, 45°, and 65° backrest angles (Fig. 14). Specifically, the muscles with significant differences were VM at 25° on both sides and right VM between 25° and 65° (*P* < 0.05). As shown in Fig. 14, all average EMG onset times occurred after 100 ms when the driver began to depress the brake pedal and did not occur after 300 ms.

## Discussion

This study provides the kinematic data and EMG of the front-seat passengers during MEB at three backrest angles of 25°, 45°, and 65°. The analysis results of these data in the corridors can reflect the biomechanical data of passengers during braking and are applicable for the development and validation of active HBMs. This data can be used as input conditions for HBM simulations to more accurately represent the real-state of passengers in pre-crash scenarios.

The EMG was normalized to MVC in isometric conditions and the typical sitting posture. Although MVCs require time-demanding isometric contraction tests for each muscle of every volunteer, it can objectively compare the normalized muscle activation levels among individuals^16–19,31^.

Reed et al.^32^ also reported the passenger head displacements during braking at backrest angles of 23°, 35°, and 47°, in which study, the average head X displacement was 181.9 mm at 23°. In another test conducted by Kai et al.^33^, the head displacement of the passenger during braking was 520 mm. In this study, the head displacement was 275.9 mm at 25°, which is reliable with previous research. The differences in kinematic data may be attributed to variations in experimental conditions. In this study, the initial vehicle speed and the jerk during braking were higher. Additionally, differences in restraint systems and seat factors may also contribute to these variations.

The backrest angle affected the passenger’s kinematics during the emergency braking, with the reclined seating increasing the forward displacement. With the backrest angle increased from 25° to 45° and 65°, the forward displacement of head increased from 275.9 to 451.8 mm and 564.8 mm, respectively; meanwhile, the displacement of T1 increased from 207.1 to 332.5 mm and 409.2 mm, respectively. The same trend has been reported by previous studies^32,34^. The seatbelt is unable to effectively constrain the torso in the reclined sitting posture, thereby increasing passenger displacement. The results presented in this study are comparable and consistent with the previous results.

The EMG in the standard sitting position showed higher activity levels and peaks than that in the study of Ólafsdóttir et al.^19^ In this study, the initial velocity was 80 km/h, and the MEB had a higher jerk. Looking more closely, the passenger head X displacement and T1 were also higher in standard seating. These factors can explain the difference in EMG between the two studies. In addition, the same muscles in the two studies showed similar curve trends. Furthermore, the longer duration for muscle activity may be due to the longer braking time by the higher initial velocity.

The muscle activation levels during the quiet sitting phase are essential because they render the muscle activity of passengers during normal driving and serve as the input for the initial state of active HBMs. Consistent with previous studies^18,19^, all muscles were activated less than 5% MVC on average. Ólafsdóttir et al.^19^ reported in quiet sitting that the average SCM activity was 1.4 and 1.6% MVC on the left and right, respectively, which is similar to this study with 1.27 and 1.49% MVC. CPVM, SERAN, and RA exhibited higher levels of activity than the other muscles. Thus, the neck muscles may be affected by the sitting posture. Östh et al.^18^ also reported that CPVM had higher activity levels during the quiet sitting. Additionally, the muscles in the torso are important to maintain the body posture. However, the higher activation levels of these muscles may be influenced by the pressure artifacts from the contact.

The change from 0 to 1 g acceleration increased the average muscle activities, and all muscles except for the TA became active. Although the study by Beeman et al.^35^ was different from this study, they compared the kinematics of volunteers and post-mortem human surrogates (PMHSs) in low-speed sled tests. They found that the average peak forward displacement of the head CG was larger for PMHSs than for the relaxed volunteers. According to the literature, the higher muscle activity may be to restrict the body motion^16^.

Following the braking maneuver, there were significant differences in the EMG peaks of the SCM on both sides at different backrest angles (*P* < 0.05). The increase in backrest angle caused higher levels of muscle activity. Therefore, the higher activation of SCM may be related to the greater displacement. Furthermore, for all backrest angles in the trials, the head displacement was larger than T1. Beeman et al.^35^ also reported greater forward head excursions relative to the torso. In summary, these results can explain the high activation levels and variations of SCM in the present study to restrict the motion of the head relative to the torso. However, a contraction of SCM may alter the resulting kinematics and injury patterns in a collision^15,36^. These effects are important using active HBMs to predict injury risk. Ghaffari et al.^16^ reported that different levels of co-contraction in muscles were most probably for maintaining the posture. For instance, the antagonistic co-contraction of SCM and CPVM helped stabilize the neck. But the same muscle activity was not observed in CPVM, and there was no significant increase in EMG activity level in the reclined position compared with the standard sitting position. It is speculated that different neck muscles may have different neuromuscular control strategies when used to stabilize the neck. However, the specific strategies for different muscles lack research.

In emergency braking, there were obvious changes in the EMG of the VM. During the steady-state phase, the average VM activity was 3.5–10% MVC. In the reclined sitting posture, VM had a higher level of activation. Tran et al.^34^ observed that the occupant had a significant forward displacement of the knee in the reclined seating, while the knee had almost no significant displacement in the standard seating. Thus, different muscle control strategies were adopted to maintain the stability of the knee with different backrest angles. Ghaffari et al.^16^ reported a similar conclusion regarding the contribution of VM to the knee stabilization. However, the contraction of the VM results in higher internal forces on the femur, which increases the risk of femoral shaft fractures^37,38^, especially in reclined sitting positions.

Although the change in acceleration increased the muscle activities, TA remained at a low activity level. The average activation peak of TA on both sides was 7.96 and 6.14% MVC at 25° and below 5% MVC for all other sitting postures. In addition, the average TA activations were below 3% MVC in all loading conditions during the steady-state phase. This result is unexpected and indicates that the support of the shank for the body during braking is limited. Especially in the reclined sitting position, it has no significant effect on limiting the forward displacement of the body.

An important finding of this study is the relationship between the activation level of the RA and the backrest angle. The RA average activation peak was 71.1% (left) and 81.7% (right) MVC at 65°, and it increased with the increase in backrest angle because the torso displacement increased in the reclined position, which might lead to higher muscle contraction. Kang et al.^21^ found that the maximum activation of RA at 32° was 2.9 ± 0.2 times higher than that in the forward-facing upright posture. Furthermore, the increased activation level of RA may be due to the higher restraint of the lap belt on the body.

There are some limitations in this study. The volunteers who participated in the trials had similar stature, weight, and BMI. Wang et al.^39^ showed that decreased abdominal injury correlated with increased subcutaneous fat thickness in crashes, but there appeared to be a trend toward worsened injury severity in the lower extremities. However, there is limited research on the muscle response of volunteers with different body sizes in potential pre-crash situations. Reed et al.^40^ found that volunteers with higher BMIs had smaller forward head excursions in the braking maneuver.

Additionally, in a small proportion of the tests, few muscles were already in an activated state at the onset of braking, but they maintained a relatively low level of activity. Reed et al.^40^ reported that the volunteers could more quickly react when they were looking directly forward in a typical passenger posture, possibly because they detected the braking motion of the driver. Blindfolds and earplugs could be used to block the visual and auditory stimuli of volunteers to eliminate the possibility of psychological expectations in future studies.

## Data Availability

All data generated or analyzed during this study are included in this published article.
